# On the Application of Friction

**Published:** 1853-01

**Authors:** Edward William Lowe

**Affiliations:** Late House-Surgeon to St. Bartholomew’s Hospital


*On the Application of Friction, as a remedy for some Diseases of the Joints. Illustrated by Cases.*—BY EDWARD WILLIAM LOWE, M. R. C. S. E. Late House-Surgeon to St. Bartholomew's Hospital. In my first trials of frictions as a remedy for diseased joints, I used it simply with a little flour, to prevent chaffering of the skin. I soon found that I had in it a powerful agent, not only by its curative powers in some cases, but also by the effect of its careless employment in others; for while, by its careful and judicious application in three or four cases, I was able to restore the joints, I learned some useful lessons by entrusting its use into subordinate and less careful hands, the results here being the induction of attacks of acute inflammation on the previous chronic disease.

My experience fully confirmed my previously-conceived opinion in favor of frictions as a remedy for diseased joints, and I determined on more extended trials for its power.

I had, from a considerable experience, been strongly and favorably impressed with the local application of ung. hydrarg, in diseases either acute or chronic of the joints; and reflection on the subject induced me to believe that a combination of the two means might prove more powerful than either individually.

The two next cases which fell under my care I accordingly rubbed with ung. hydr., in lieu of the simple flour. The benefit was marked; the cure was expedited, and without any general mercurial effect being produced. Knowing the effect of general and equable pressure in aiding the restorative action of diseased parts, and in promoting absorption, I determined on employing its powers also in aiding the object I had in view.

This combination of means has proved a powerful union, satisfactory in the highest degree, as I trust the following cases will prove.

Before, however, I proceed to describe the cases, I will briefly explain the manner of applying the remedy.

The joint having been carefully examined, and considered one fit for the remedy, if painful or tender of pressure, I take it and gently and easily rub it with ung. hyd. for a few minutes, avoiding as much as possible to give pain. Having done this, I wrap a piece of lint around the joint, now covered thickly with the ointment, and then smoothly apply straps of plasters for some distance below to the same distance above the joint, being careful that the sustaining pressure be equable, and at the same time slight. When finished the joint should feel easy, supported rather than pressed, certainly not in any part unduly pressed.

These dressings I allow to remain on four days or a week; they are then removed, and the same process repeated, if the joint will tolerate it, the friction being stronger and longer continued. Thus I go on, removing the dressings; and each time as the joint will bear it, applying more and more powerful friction, until it is downright hard and quick work for the rubber; and afterwards, each time making the pressure greater and greater, until the strapping can be comfortably borne as tight as it can be put on.

The great art in the application of the remedy being to, at first, so gently rub the joint, and so easily press it with the strapping, that as little pain as possible shall be given. A little pain you will give by the friction; it should, however, be but little, or, as a consequence, the joint will soon become hot, red, more swollen, and painful—in short, acutely inflamed, which condition will, of course, require removal by the appropriate remedies before you can resort to the friction.

If, however, attention be paid to this point, and to the easy application of the strapping, so that, when finished, the joint shall feel comfortable, it will soon become tolerant of increased frictions and tighter pressure, and the result will be, that, in a short time, a joint will bear hard rubbing and tight pressing, which when you first began, was painful even to gentle handling.

The one great caution I give is against a desire to do too much. Be content to advance slowly, and you will do so surely ; attempt to push too eagerly forwards, and the chances are you will throw yourself backwards. Never continue the friction if it is painful, or apply the pressure so tightly as to cause pain ; then you will find your joint rapidly become tolerant of the friction and pressure.

The strapping I use is emp.-robor., spread on moleskin. I find it more adhesive, and at the same time more supple, than simple adhesive. It should be cut into straps half an inch wide, and should be neatly applied, one a little over-laying the preceding one, so as entirely to cover the joint.

The friction, gradually increased from a little, may at last be made as firmly and quickly as can be done, for the space of half an hour—a longer period is seldom safe.

Towards the latter end of the cases, when the disease was well-nigh mastered, I have usually substituted a stimulating liniment, in lieu of the ung. hydrarg., and have believed that, by so doing, the end I desired was expedited.

*Theory of its Action.*—It is difficult clearly to prove it. I am inclined, however, to attribute to it the property of increasing the power of the circulation through the parts, and of stimulating the action of the absorbents; so that, while a more healthy vigour is given to the blood-vessels of the parts, the absorbing vessels at the same time are excited to a stronger action. This may be true, or it may not. It is, perhaps, equally difficult to prove or gainsay it. But, be it true or be it not, the fact of the efficacy of the plan of treatment remains the same.

The action of the local application of mercury is probably, as Mr. Scott long since described, in giving tone to the vessels of the part, without at the same time producing any general mercurial effect. In this respect its action is, I believe, powerful and peculiar—essentially specific.

The pressure, at first, only acts as a means of support to the weakened vessels of the part; in this way acting as powerful auxiliary to the restoration of a healthy action in them. The subsequent pressure, however, which I apply as the joint becomes able to bear it, acts, I believe, as all pressure does, by inducing absorption.

Thus, then, the three means employed are all exerting their energies in one direction; and, while we are observing them, let us not overlook another effect they produce, viz., that of maintaining the diseased parts at a moderate and regular temperature. This is of the utmost importance; for it is, I believe, of itself capable of restoring some joints, while, on the contrary, I feel confident that, without attention to it, none will get well, be the other means used what they may. Mr. Tamplin's remarks on this subject will be borne out by all who have much to do with diseases of the joints. The firmness and stiffness of the strapping while it produces the pressure you desire, acts at the same time as a well-fitted splint, preventing much motion of the joint. The usefulness of this is so evident as to require no comment.—*Canada Med. Jour.*

